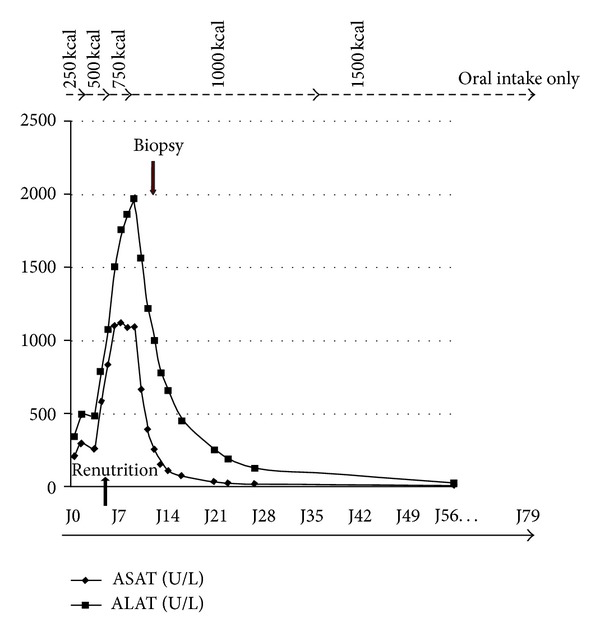# Severe Starvation-Induced Hepatocyte Autophagy as a Cause of Acute Liver Injury in Anorexia Nervosa: A Case Report

**DOI:** 10.1155/2013/749169

**Published:** 2013-07-30

**Authors:** S. Restellini, L. Spahr, L. Rubbia Brandt

**Affiliations:** ^1^Department of Specialties, Division of Gastroenterology and Hepatology, Geneva's University Hospitals and University of Geneva, Gabrielle-Perret-Gentil 4, 1205 Geneva, Switzerland; ^2^Department of Genetic and Laboratory Medicine, Division of Clinical Pathology, Geneva's University Hospitals and University of Geneva, Gabrielle-Perret-Gentil 4, 1205 Geneva, Switzerland

## Abstract

*Introduction*. Mild elevation of transaminase may be observed in anorexia nervosa, but acute liver injury is uncommon. A complex programmed cell death in response to starvation, called autophagy, has been described in experimental and human studies. *Case Presentation*. A 24-year-old woman suffering from anorexia nervosa was hospitalized for severe malnutrition. At admission, there were biological signs of acute liver injury but no electrolytic imbalance. After having ruled out the most common causes of liver injury, the patient was carefully refed. As liver tests remained abnormal, liver biopsy was performed. At histology and electron microscopy, numerous signs suggestive of starvation-induced hepatocyte autophagy were found. *Discussion*. Severe starvation can be associated with acute liver injury that is slowly reversible with careful enteral nutrition. In this clinical situation, profound hepatic glycogen depletion in association with autophagy appears as the leading cause of liver injury.

## 1. Introduction

Anorexia nervosa is a common eating disorder, particularly in women, with a lifetime prevalence estimated to be 0.3–0.9% [1]. This disorder is associated with numerous medical complications (cardiovascular problems, endocrine disorders, electrolyte and hematopoietic abnormalities, amenorrhea, and osteoporosis), among which changes in liver tests are frequent. A mild increase in serum aminotransferase levels is indeed observed in up to 60% of these patients [2, 3] leading the American Psychiatric Association to recommend a systematic assessment of liver tests for these patients [4]. Conversely, severe liver damage with a significant decrease in coagulation factors appears to be uncommon in patients with anorexia nervosa, although some cases have been reported [5]. Even if the mechanisms of liver injury during anorexia nervosa remain unclear, a complex programmed cell death in response to starvation, called autophagy, has been suggested in few studies to play a pivotal role in yeast and animals [6] as well as in human beings [7]. We report here the case of a 24-year-old woman with anorexia nervosa, who experienced acute liver injury in association with histological and electron microscopical evidence of liver autophagy that was slowly reversible following careful renutrition.

## 2. Case Presentation 

A 24-year-old woman was admitted for severe malnutrition and dehydration in the context of anorexia nervosa that was diagnosed one year ago. During this time, she had been hospitalized during 4 months with a transitory gain of six kilograms. She was treated with serotonin reuptake inhibitor and denied taking illicit drug or potentially harmful products. On admission, she presented hypotension (90/50 mmHg), sinus bradycardia (40 bpm), slight hypoglycemia (3.9 mmol/L), and hypothermia (35.7°C). She measured 160 cm and her body weight was 36 kg, corresponding to a body mass index (BMI) of 14 kg/m^2^. At physical examination she was oriented but lethargic and slightly jaundiced. The abdomen was slightly tender without ascites. The liver was not enlarged.

Laboratory data at admission demonstrated the following: glycemia (3.5 mmol/L), Na^+^ 137 mmol/L (N: 136–144), K^+^ 3.7 mmol/L (3.6–4.6), total Ca^2+^ 2.34 mmol/L (N: 2.2–2.52), Mg^2+^ 1.10 mmol/L (N: 0.65–1.05), P^2−^ 1.22 (N: 0.8–1.5), and osmolality 289 mOsm/kg. Platelets were at 144 G/L (N: 150–350), hemoglobin was at 133 g/L (N: 120–160), and leucocytes were at 6.6 G/L (N: 4–11). The liver function tests was abnormal with a factor V at 33% (N: >70%). Serum aminotransferase levels were increased as follow: AST 208 U/L (N: 11–42), ALT 341 (N: 9–42), and total bilirubin 65 *μ*mol/L (N: 7–25). The vitamin C was normal at 78 umol/L (N: 17–85) as was the thiamine at 208 umol/L (N: 65–200).

She was treated with serotonin reuptake inhibitor and denied taking illicit drug or potentially harmful products. Her treatment was stopped, but laboratory tests continued to deteriorate. 

After one week, the laboratory routine tests revealed abnormal hematological tests with hemoglobin at 114 g/L, leucopenia at 2.5 G/L, and thrombocytopenia at 52 G/L. Additional investigations included prealbumin: 121 mg/L (N: 223–380) and albumin: 36 g/L (N: 35–48). Electrolytes remained normal. Renal and thyroid functions were unremarkable. Liver tests were as follows: AST 1088 U/L, ALT 1868 U/L, GGT 138 U/L, PA 102 U/L, and total bilirubin 66 *μ*mol/L. INR was at 1.02 and factor V at 85%. Additional laboratory tests were performed to exclude all possible causes of liver injury. Hepatitis A, B, C, and E, CMV, and HIV tests were negative. Autoantibodies were absent; iron studies and ceruloplasmin were normal. At abdominal ultrasound the liver appeared normal with no ascites. Portal and hepatic veins were unremarkable. Thus, a diagnosis of acute liver injury associated with anorexia nervosa was considered the most plausible diagnosis. 

A careful and progressive refeeding was performed, but liver transaminases remained abnormal. Thus, 10 days after hospital admission, a liver biopsy was performed by the transjugular route.

**Figure 1:**
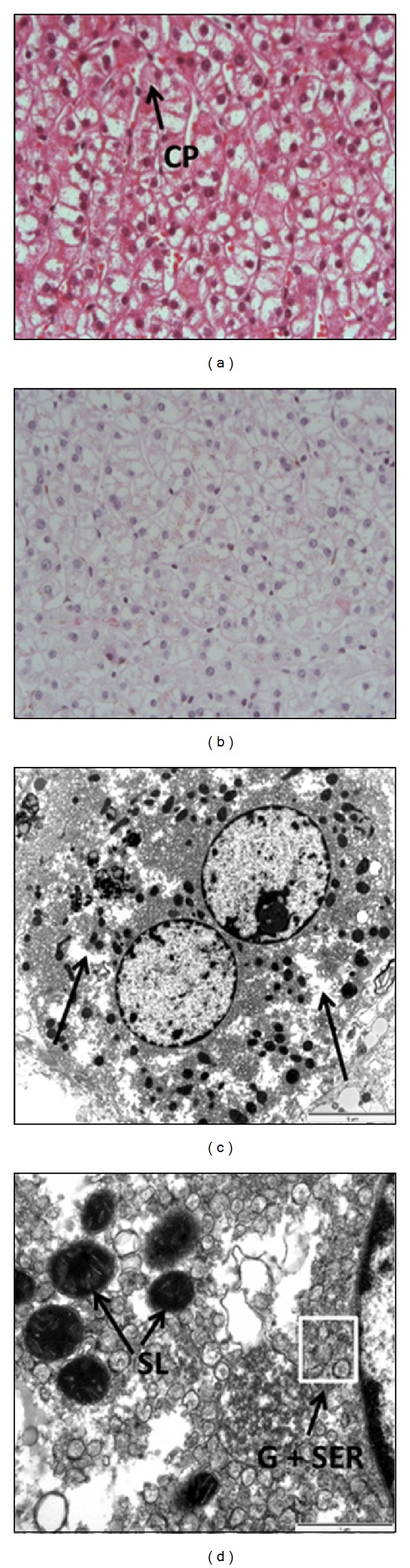
Histology and electron microscopy. (a) Normal liver lobule with pale hepatocytes and ceroid pigments (CP) at their apical pole (H&E staining, original magnification ×400). (b) Diffuse clarification of hepatocytes in relation with marked glycogen reduction (PAS staining). (c) Electron microscopic appearance of a typical hepatocyte showing glycogen deficit (white areas). (d) Overview of the cytoplasm of a typical hepatocyte at electron microscopy. Low density of rough endoplasmic reticulum, mitochondria, and primary lysosomes. Increased number and hyperplasia of smooth endoplasmic reticulum (SER) and Golgi (G) apparatus (white box). Secondary lysosomes (SL) considered in that case as autophagosomes.

At histology, architecture of the liver was normal without fibrosis. Portal tracts and biliary and vascular structures were normal. Cytoplasm of hepatocytes was clarified and some acidophil bodies were found predominant in the centrilobular region. In addition, ceroid pigments at the apical pole of the hepatocytes were observed (Figure 1).

An electron microscopy study was also performed in a fragment of liver biopsy, which showed features that strongly suggested a phenomenon of autophagocytosis: the density of glycogen particles in the hepatocytes was very low, consistent with cytoplasm clarification on PAS staining at the optical microscopy. In addition, the number of both mitochondria and rough endoplasmic reticulum appeared to be reduced. This observation contrasts with an increased number of Golgi bodies and smooth endoplasmic reticulum as well as primary and secondary lysosomes containing digested material (Figure 1). Eight weeks after admission, with progressive refeeding the test returned back to normal (Figure 2).

## 3. Discussion

This case of anorexia nervosa illustrates the consequences of severe malnutrition on the liver, with marked elevation of liver enzymes, profound glycogen depletion, and histological evidence of autophagocytosis. Although mild liver injury is not rare and is reported in up to 60% of patients with anorexia nervosa, high levels of serum transaminases are rather uncommon [3, 8]. A median peak value of ALT of 56 times normal has been reported in a recent case series. This biological alteration has been found to inversely correlate with the body mass index, suggesting that severe starvation is involved in the pathogenic mechanism of anorexia nervosa associated liver injury. Surprisingly, these biological signs of acute liver injury contrast with the absence of liver cell necrosis, which we would expect with such an elevation in liver enzymes [9].

Accordingly, Rautou et al. have described in the majority of their cases histological alterations including swelling of hepatocytes with cytoplasmic clarification and ceroid pigments, in the absence of inflammatory changes and fibrosis [7]. Similar changes were observed in our patient. Overall, these histological findings are consistent with severe glycogen depletion as the primary cause of liver injury. A more detailed examination of liver tissue using electron microscopy brings additional information on the consequences of severe starvation. In addition to low density of glycogen particles, we observed a decreased number of mitochondria and endoplasmic reticulum together with an increase in autophagosomes, similar to the findings of a recent study [10]. These alterations illustrate the consequences of autophagy, which is a physiological mechanism that the cell activates during episodes of stress including severe starvation. During this process, cytoplasmic components are degradated and appear as autophagosomes at electron microscopy studies, as in the present case. Therefore, it is tempting to speculate that the elevated transaminases originate from the altered mitochondria. In conclusion, severe starvation associated autophagy can be regarded as a “self-cannibalism” action of the organism to cope with this stressful condition [10]. It can be associated with acute liver injury that is slowly reversible with careful enteral nutrition. In this clinical situation, profound hepatic glycogen depletion in association with autophagy appears as the leading cause of liver injury. 

## Figures and Tables

**Figure 2:**